# Multiple Criterion and Multiple Stimulus Signal Detection Theory Analysis of Corneal Painful and Cool Pneumatic Stimuli

**DOI:** 10.3389/fphar.2022.759748

**Published:** 2022-03-18

**Authors:** Varadharajan Jayakumar, Trefford Simpson

**Affiliations:** School of Optometry and Vision Science, Faculty of Science, University of Waterloo, Waterloo, ON, Canada

**Keywords:** cornea, pain, psychophysics, human, signal detection theory

## Abstract

**Purpose:** To evaluate the detectability of pneumatic corneal stimuli and response bias using multi-stimuli multi-criterion signal detection theory (MSDT).

**Methods:** Thirty-six participants were recruited using convenience sampling. A Waterloo Belmonte esthesiometer was used to deliver cold, mechanical, and chemical stimuli to the center of the cornea at three separate study visits. The stimulus type was assigned randomly to each visit at the start of the study. The threshold (baseline for detection theory experiment) for the assigned stimulus type was obtained using the ascending method of limits. In the cold and mechanical MSDT experiments, 100 trials (80 signal (20 each for 4 intensities) and 20 catch trials) were presented in randomized order, and participants responded with a 5-point confidence rating to each trial. In the chemical MSDT experiments, 50 trials (20 signal trials each for two intensities and 10 catch trials) were presented, and responses were provided using 4-point confidence ratings. Detection theory indices were calculated individually and as groups, which were then analyzed using mixed models and paired t-tests.

**Results:** Detectability (d_a_) and the area under the curve (A_z_) were significantly different between stimulus intensities within each stimulus type (all *p* < 0.001) but were not different between the stimulus types. Receiver operating characteristics (ROC) curves were separable between the scaled intensities for all stimulus types, and no overlaps were observed in the z-ROC space. The log-likelihood ratio (*lnβ*) depended on stimulus intensity and psychophysical criterion for all stimulus types.

**Conclusion:** It is feasible to use MSDT for analyzing ocular surface sensory processing and the theory provides insight into the possible bias associated with the use of pneumatic stimuli. With noxious and non-noxious pneumatic stimulation, detectability and criteria vary systematically with stimulus intensity, a result that cannot be derived using classical psychophysics and this highlights the importance of signal detection theory and its approaches in studying ocular surface pain and thermal processing.

## Introduction

The corneal neurons are classified into Aδ-fibers (thinly myelinated and fast conducting) and C-fibers (unmyelinated and slow conducting) based on the thickness of the myelin sheath surrounding them and their conduction velocities, which transmits impulses from cornea to trigeminal ganglion and farther to the brain for pain processing (Tanelian and Beuerman, 1984; Belmonte et al., 1991; MacIver and Tanelian, 1993a; Gallar et al., 1993; Chen et al., 1995; Kovács et al., 2016). Three types of corneal receptors (polymodal nociceptors, mechano-nociceptors, and cold receptors) have been identified electrophysiologically in non-primates, which detect the signal and transmit impulses either through Aδ or C-fibers (Belmonte and Giraldez, 1981; Tanelian and MacIver, 1990; MacIver and Tanelian, 1993b; Belmonte et al., 1997; Müller et al., 2003). The cold thermo-receptors and polymodal nociceptors transduce signals conducted through the C-fibers, while the mechano-nociceptors transduce information for the fast-conducting Aδ-fibers’ rapid response to painful mechanical stimuli (MacIver and Tanelian, 1993a; MacIver and Tanelian, 1993b; Belmonte et al., 2004). Since there is no systematic neurophysiological examination on the effects of human corneal stimulations, the presence of receptors/channels in the human cornea has been evaluated psychophysically (Feng and Simpson, 2004; Jayakumar and Simpson, 2020). Feng and Simpson (2004) have identified multiple corneal psychophysical channels in the human cornea. Our previous report using signal detection theory (SDT) showed favorable evidence in our data toward both the nerve conduction and nociception hypotheses (Jayakumar and Simpson, 2020).

The detection of the human ocular surface stimuli is complex due to the interdependence of the components of the ocular surface sensory processing system (both within and between the cornea and conjunctiva) (Feng and Simpson, 2004; Feng and Simpson, 2005). Detection thresholds estimated using classical psychophysical methods have been used as a measure of ocular surface sensory processing, even though they have been found to vary (Murphy et al., 1996; Acosta et al., 2001; Feng and Simpson, 2003; Golebiowski et al., 2005; Situ et al., 2008; Golebiowski et al., 2011). Variable observer’s decision criteria are a major influence on threshold measurements (Swets, 1961; Gescheider, 1997) and these may lead to biased decisions by observers. Examples producing these biases include time of the experiment, previous experience and training, instruction characteristics, signal probability, stimulus intensity, or presumed tolerability to pain (Swets, 1961; Chapman, 1977; Rollman, 1977; Vision, 1985; Gescheider, 1997; Macmillan and Creelman, 2005). Only 1 experimental investigation of ocular surface sensory and decision criteria derived using signal detection theory (SDT) has ever been published (Jayakumar and Simpson, 2020). In it, we showed among other things, that there was a shortcoming in understanding the criteria used by participants because the simple yes-no experiment was designed to examine only the single criterion used by each subject (Jayakumar and Simpson, 2020).

The yes-no SDT experiment involved a detection task, in which participants detected the presence of a signal (supra-threshold stimulus) against the background noise. The yes-no SDT experiment demonstrated the feasibility of using one-interval two response (yes-no) design SDT to analyze the ocular surface sensory processing (OSSP) of pneumatic stimuli. However, there were a few limitations in the experiment that needed to be addressed, such as the assumption of fixed criterion, detection indices obtained only for a single intensity, and longer experiment duration if we need to test each intensity separately in a similar protocol. Yes-no SDT assumes that participants use a single criterion throughout the experiment when responding “Yes” or “No” to a trial, similar to the assumed single (and fixed) criterion in a classical psychophysical method but with the ability to estimate bias (Green et al., 1974; Gescheider, 1997; Macmillan and Creelman, 2005). However, if the participants vary their criterion during the experiment, the variation cannot be distinguished/evaluated due to the two-response design. Pay-off matrices or changes in instructions provided before the experiment have been reported in the literature to control/alter the criterion assumed by the participants (Green et al., 1974; Gescheider, 1997; Macmillan and Creelman, 2005). However, these restrict the participants from choosing their criterion independently during the experiment. Also, in a normal/clinical/experimental environment, the cornea receives multiple stimuli of different types and intensity at the same time. For example, in a clinical environment, participants may have to detect the stimuli of different intensities while they are already experiencing discomfort from the pre-existing dry eyes or factors such as drafts and dry air conditioning (Mendell and Smith, 1990; Wolkoff et al., 2005). These limitations make the yes-no one-interval SDT design less efficient, but the flexibility of SDT is that the same experiment could be conducted with variable criteria and multiple stimuli instead of a single stimulus intensity yes-no design. SDT experiments with variable criteria are usually referred to as multi-criterion or rating SDT experiment and in rating SDT experiments, instead of reporting a yes/no detection response, participants rate their confidence with which they detected a signal compared to the background noise (Green et al., 1974; Gescheider, 1997; Stanislaw and Todorov, 1999; Falmagne, 2002; Macmillan and Creelman, 2005; Wickens, 2010). Each level is then “converted” to a yes-no design to obtain different criteria adopted by the participants during the experiment, which will be similar to conducting multiple yes-no experiments with different pay-off matrices. Either ends of the rating scale (1 and 5, if 1-5 rating scale is used) represent the most conservative or most lax criteria used by the participants during the experiment, but participants can independently choose and vary their criterion during the experiment (Green et al., 1974; Gescheider, 1988; Stanislaw and Todorov, 1999; Wickens, 2010). Also, the detection indices may be estimated for multiple intensities within a single rating SDT experiment and here we refer to this as multi-stimulus rating SDT (MSDT). (Green et al., 1974; Gescheider, 1988).

MSDT experiments with pneumatic stimuli have never been conducted to examine OSSP. In the only previously reported OSSP study using MSDT, detectability of thermal waterjet corneal stimuli was obtained from rating responses, but the results were reported as though the experiment was conducted as a yes-no SDT experiment (Beuerman and Rozsa, 1985). MSDT has been used in many other areas such as audition, memory, and pain (Clark and Mehl, 1973; Green et al., 1974; Gescheider, 1988; Belmonte et al., 1997; Macmillan and Creelman, 2005; Weidemann and Kahana, 2016). We initiated a series of signal detection theory approaches to understanding OSSP because of its similarity to somatic pain processing instead of using the trigeminal pathway, and signal arising from similar pain receptors (Millodot, 1984; Müller et al., 1995; Belmonte and Cervero, 1997; Müller et al., 2003; Belmonte et al., 2015; Belmonte et al., 2017).

According to the International Association for the Study of Pain, pain is an “unpleasant sensory and emotional experience associated with actual or potential tissue damage or described in terms of such damage” (Bonica, 1979; Merskey, 1994), and recently Williams and Craig (2016) defined pain as “a distressing experience associated with actual or potential tissue damage with sensory, emotional, cognitive, and social components.” Studies have found that psycho-social entities such as anxiety, fear, personality, confidence, decision-making, self-esteem, and stress affect the perception of painful stimuli (Leventhal and Everhart, 1979; Frenkel et al., 2009; de Visser et al., 2010). Similar issues have been suggested in the literature of corneal sensitivity (Millodot, 1984), but have never been addressed before.

According to SDT, to elicit a response for a given trial, the sensory process first detects the stimulus and this is then followed by the decision process (influenced by multiple factors) that shifts the response either in favor of signal or noise (Green et al., 1974; Wickens, 2010). Both the sensory and decision process can be measured simultaneously and independent of each other using SDT. So, the aim of this experiment was to evaluate the feasibility of using MSDT to understand the OSSP of corneal pneumatic stimuli. This paper is primarily a technical report dealing with a complex issue affecting the psychophysical measurement of ocular surface sensing.

## Methods

Forty-one participants were recruited in the study using convenience sampling from the students and staff community of the University of Waterloo. The study was conducted according to the guidelines of the Declaration of Helsinki and ethics approval was obtained from the University of Waterloo, Office of Research Ethics (Waterloo, Ontario, Canada). Informed consent was obtained from each participant and participants were allowed to discontinue at any stage of the study. The ocular surface was screened for any active signs of inflammation or infection. There were only soft contact lens wearers in this study and the lens wearers were instructed not to wear their contact lenses on the day of their study visits. The visits were scheduled to occur at the same time of the day (±30 min) to reduce diurnal variation affecting the measurement.

### Sample Size

Since this MSDT experiment using ocular surface stimuli has never been performed before (Jayakumar and Simpson, 2020), we used the data from our Yes-No experiment (Jayakumar and Simpson, 2020) to calculate the sample size for this experiment using the gpower 3.1.9.6. The estimated sample size needed was 8 (two-sided pair *t*-test, alpha = 0.05, beta = 0.8) with effect size of 1.17 based on the mean ± SD of cold and mechanical stimuli.

### Stimulus Characteristics

The stimulus types used in this experiment were mechanical, chemical, and cold (or cool, room temperature). A Waterloo Belmonte pneumatic esthesiometer was used to deliver each stimulus to the center of the anterior corneal surface. The mechanical stimulus was medical air, heated to 50°C (converts to 33°C at the corneal surface) at the nozzle, and the cold stimulus was a room-temperature medical air. The flow rate of the stimulus was either increased or decreased to alter the intensity of the output, depending on the response provided by the participants. In the case of the chemical stimulus, the flow rate of the stimulus was kept constant at half of the mechanical threshold to remove any mechanical effect influencing the judgment. The ratio of carbon dioxide mixing with the medical air was changed at a given flow rate to produce a chemical stimulus. The % CO_2_ in the stimulus defines the intensity of the chemical sensation induced. The flow meters in the control box of the esthesiometer regulate the flow of medical air and CO_2_ to the desired concentration and flow. The temperature of the chemical stimuli was the same as the mechanical stimuli. The preparation and delivery of the stimulus were automated using the custom software according to the psychophysical procedure conducted. Each stimulus type was randomly assigned to one of the three study visits at the start of the first study visit. Each visit was approximately 1 h long and was separated by at least a day to avoid fatigue effects and allow ‘recovery’ of the ocular surface and the pain processing system.

### Ascending Method of Limits to Determine Threshold

Though it is an MSDT experiment, the detection thresholds were calculated to use as a baseline for the following MSDT experiment. At the start of the visit, detection thresholds for the assigned stimulus were measured using the ascending method of limits (AMOL). An average of three measures was considered as a threshold. The duration of the chemical stimulus was 2 s, and mechanical and cold stimuli were 3 s long. The inter-stimulus interval for cold and mechanical stimuli was 10 s; for chemical stimuli, the inter-stimulus interval was 30 s (to enable purging of the stimulus in preparation for the subsequent stimulus). The oral instructions were provided by the examiner before the start of the experiment, followed by the automated audio prompts for each trial. The training was provided. Participants were advised to blink between each trial. Participants responded yes/no to each trial using the button box and the responses were recorded in the software. If the difference in detection thresholds between 3 measures was larger than 15 ml/min or 15%, the experiment was repeated another day. If the thresholds were still variable, the participants were excluded from the study.

### Detectability Experiments

**FIGURE 1:**
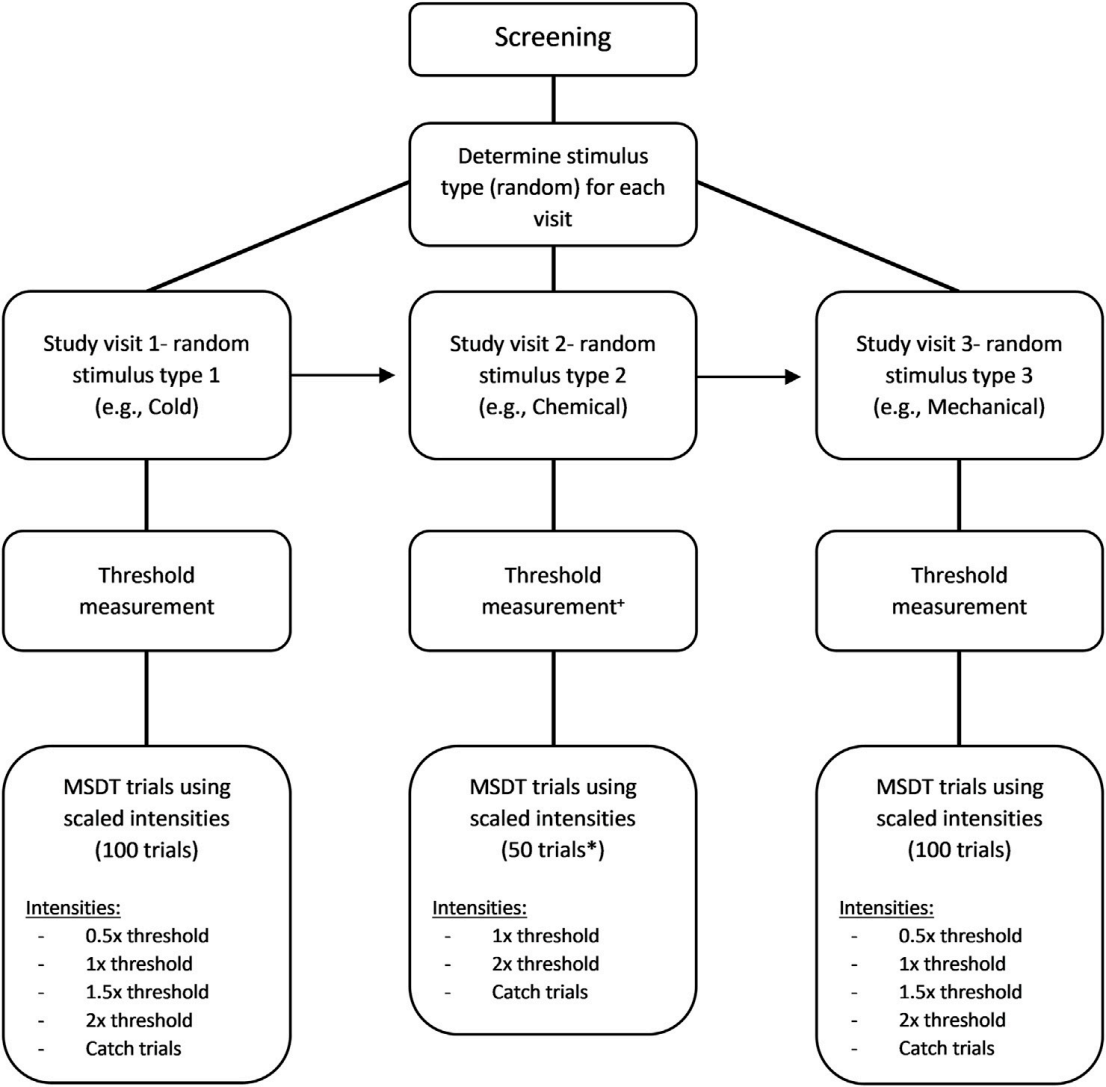
Flow chart of the MSDT experiment. The order used in this example is cold (first visit), chemical (second visit), and mechanical (third visit) stimuli. ^+^For chemical threshold measurement, mechanical threshold was measured first followed by the chemical threshold with half of the mechanical threshold as the flow rate of chemical stimuli. * 50 trials were used only for chemical MSDT experiments.

The signal intensities for the MSDT experiments were scaled based on their respective corneal detection thresholds and the signals (in the analysis and report) were referred based on relative intensity to the threshold (Figure 1). The scaled intensities were described later in the methods. Instructions for the detectability experiment were accompanied by a short demonstration of the trial sequence. ‘Neutral’ instructions were scripted and delivered to all participants at the start of the experiment, to minimize examiner induced bias and variability. The stimulus probabilities and feedbacks, indicating the correctness of the response were not provided to the participants. Instead, audio feedback confirmed each button press. Participants rated each trial using the button box and the number of button presses was stored as the rating for each trial. Participants were advised to blink between stimulus presentations.

### Cold and Mechanical Detectability Experiments

The cold and mechanical MSDT experiments consisted of 100 trials with random presentations of a signal or a noise stimulus (Figure 1). Each experiment consisted of four signal intensities of 20 trials each and a noise stimulus of 20 trials. The signal intensities (scaled based on detection thresholds) were a sub-threshold (0.5× threshold), a threshold, and two supra-threshold (1.5× and 2× threshold) intensities. The noise stimulus was a catch trial with no stimulus. If the estimated threshold for cold or mechanical stimulus was between 15 ml/min and 20 ml/min, a flow rate of 10 ml/min was used as the intensity of the sub-threshold stimulus. If the threshold was below 15 ml/min, the trials involving sub-threshold stimulus were replaced with the blanks (catch trials) as the flow rate of 50% threshold would be well below the esthesiometer’s reliable output range of 10–200 ml/min. On a given trial, either a signal (one of the four scaled stimulus intensities) or a noise (blank stimulus) trial was randomly presented, and the instructions for the noise trials were exactly the same as the signal trials.

The inter-stimulus interval and presentation time was the same as the threshold experiment. A confidence rating scale of 5 ratings was used by the participants to respond to each trial (Table 1). Breaks were provided after 50 trials by default or whenever participants pause the experiment using a button box.

**TABLE 1:** The confidence rating scale used by the participants to respond to a mechanical or a cold stimulus trial.

1	2	3	4	5
*Definitely “No”* signal was not presented	*Probably “No”* signal was not presented	Not sure/uncertain	*Probably “Yes”* a signal was presented	*Definitely* “*Yes*” signal was presented

### Chemical Detectability Experiment

In order to keep the duration of this phase of experimentation approximately the same as those for mechanical and cold, we used the following protocol: The chemical MSDT experiment consisted of 50 trials with random presentations of either a signal or a noise stimulus (Figure 1). There were two signal intensities (the threshold and the 2x threshold) of 20 trials each and 10 noise trials. Unlike cold and mechanical MSDT experiments, the noise/catch trials for chemical stimuli were not completely blank stimuli; instead, a medical air stimulus with 0% CO_2_ was added at the same flow rate as signal trials. A confidence rating of 4 ratings was used by the participants to respond to each trial (Table 2). Breaks were provided after 25 trials by default or whenever participants pause the experiment using a button box.

**TABLE 2:** The confidence rating scale used by the participants to respond to a chemical stimulus trial.

1	2	3	4
*Definitely “No”* signal was not presented	*Probably “No”* signal was not presented	*Probably “Yes”* a signal was presented	*Definitely* “*Yes*” a signal was presented

### Data Analysis

The rating data for each participant was exported to a Microsoft Excel spreadsheet. The RscorePlus software (v.5.6.1)^49^ was used to calculate the detection theory parameters. These were based on assumptions of Gaussian signal and noise distributions. The RscorePlus data input file had the information on the number of rating categories, the number of signals (including catch trials), participant id, commands specific for SDT analysis along with the response frequency for each rating category. The commands included code for collapsing data in case of unsuccessful analysis, treatment of zero frequencies, and type of the SDT experiment. For this study, the SDT indices were calculated with an SINT (single-interval experiment paradigm) SDT protocol and zero frequencies were replaced with 1/number of rating categories to eliminate errors due to zero frequencies. The hit rate (HR) and false alarm rate (FAR) were calculated by cumulating the rating responses of n ratings for (n-1) decision criteria similar to the yes-no procedure. The HR and FAR were used in the calculation of detection theory parameters such as detectability (d’ or d_a_) and criteria. The outputs included the detection theory parameters for each signal and formatted datasheet for creating detection theory graphs using R. The equations used in calculating each detection theory parameter as provided by the software manual are listed below (Harvey, 2010):

$$d^{\text{'}}=z\left(HR\right)-z\left(FAR\right)\;\left(\mathrm{Equal}\;\mathrm{variance}\;\mathrm{model}\right)$$

$$d_{a}=\sqrt{\frac{2}{1+b^{2}}}\;.\left(z\left(HR\right)-b.z\left(FAR\right)\right)\;\left(\mathrm{Unequal variance model}\right)$$

$$A_{z}=z^{-1}\left[\frac{d_{a}}{\surd 2}\right]$$

c=−0.5 (z(HR)+z(FAR))

$$\text{ln}\left(\beta \right)=\frac{\left[z{\left(FAR\right)}^{2}\right]-\left[z{\left(HR\right)}^{2}\right]}{2}$$

**FIGURE 2:**
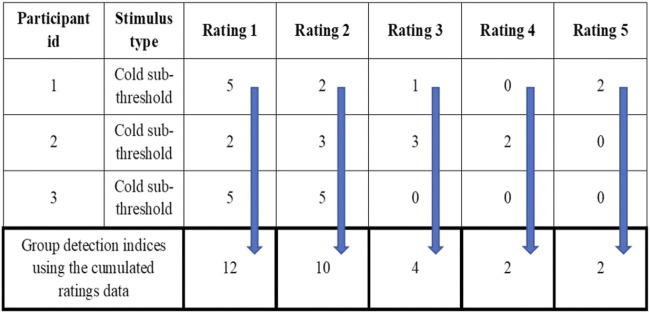
Example for the cumulated ratings to calculate group detection indices and draw group ROC curves.

The d_a_ provides the distance between the means of signal + noise distribution and noise distributions indicating the ability of subjects to detect signal from the background noise. The d_a_ and d’ are numerically the same if the variance of the Gaussian distribution of noise and signal + noise are the same (Harvey, 2010; Harvey, 1992). The A_z_ provides the area under the curve estimate for each signal. The criteria (c and *ln*β) give independent bias indices for each stimulus intensity used inside the MSDT experiment. The receiver operating characteristics (ROC) curves were plotted for individual and cumulated (grouped) data. The cumulated data ROC curves were plotted using the rating data obtained by adding the response frequencies of each stimulus rating category across all the participants within the group as though a single participant received all the trials (Figure 2). For example, all 3600 trials (720 catch and 2880 signal trials) for mechanical stimuli were received by a single participant compared to 100 trials each by 36 participants. The R programming codes provided in the RscorePlus software package (Harvey, 2018) were used in plotting the ROCs, zROCs, and Gaussian distributions.

To analyze the bias between the types of stimuli, the multiple criterion data from the rating dataset were collapsed to a single criterion yes-no type analysis due to the difference in the rating scales between the stimulus types used by the participants to respond to the trials. The ratings were accumulated based on “liberal” and “strict” criteria. In the case of the “liberal criterion”, a rating of 1 (definitely “no” there was no signal presented) was used as the frequency of “no” responses and ratings of more than 1 were accumulated as the frequency of “yes” responses which would be similar to criterion 1 from the rating analysis. In the case of the ‘strict criterion’, a rating of 5 (definitely “yes” there was a signal) was used as the frequency of “yes” response (rating 4 for chemical stimuli) and the ratings of less than 5 were cumulated as the frequency of “no” responses which would be similar to criterion 4 (criterion 3 for chemical) from the rating analysis.

The detection theory indices were analyzed using a mixed-model analysis of variance (mixed-model ANOVA) (“lmerTest” package (Kuznetsova et al., 2017)) and paired sample *t*-test in R. The post-hoc/contrast analysis for the mixed models was performed using the “psycho” package (Makowski, 2018a). Several R packages were used in sorting, rearranging and analyzing data, and in creating and exporting graphs (Lemon, 2006; Wickham, 2007; Wickham and Winston, 2011; Xie, 2012; Hope, 2013; Bates et al., 2015; Wickham, 2016; Kuznetsova et al., 2017; Makowski, 2018b; Pinheiro et al., 2019; Wickham and Henry, 2019; Wickham and readxl, 2019; Kassambara, 2020; Wilke, 2020; Harrell, 2021; Manuilova and Andre Schuetzenmeister, 2021; Revelle, 2021; Wickman et al., 2021). An alpha value of *p* ≤ 0.05 was assumed to be significant in all the analyses conducted.

## Results

The mean (±SD) age group of the participants was 30 ± 7.44 (range: 19–50) years. Five participants were discontinued at different stages of the study: Three discontinued due to variable detection thresholds obtained while repeating the AMOL and 2 participants discontinued due to high threshold. As mentioned earlier, the detection theory indices for all participants were calculated in two formats: 1) calculated using the cumulated rating data (for each rating) and 2) calculated from each participant’s rating data. The average detection thresholds for cold, mechanical, and chemical stimuli were 26 ± 2.10 (ml/min at room temperature), 29 ± 2.25 (ml/min at corneal temperature), and 25 ± 2.30 (%).

Comparisons of detection theory indices between stimulus types follow.

### Detectability

**FIGURE 3:**
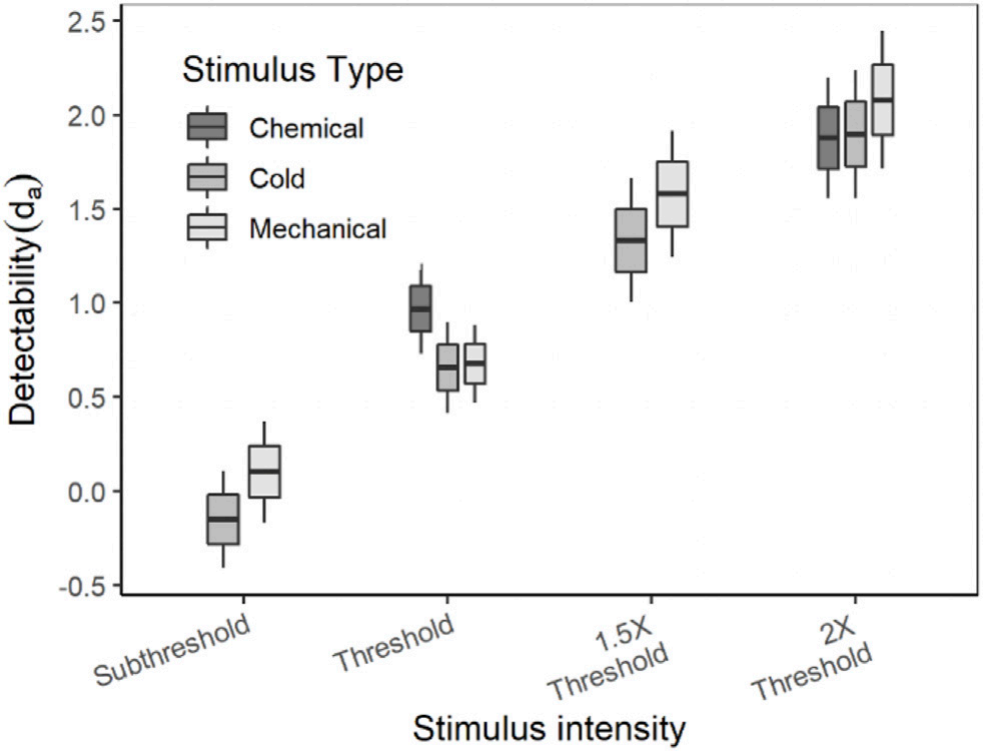
Comparison of d_a_ between stimulus intensities and stimulus types.

The average (±SE) d_a_ of each stimulus type and intensity are listed in Table 3. As mentioned earlier in the methods, the stimuli for detection theory experiments were scaled based on the threshold and the term “threshold” in detection theory experiments is used to indicate the intensity of the stimulus and not the outcome of the experiment. Since the detection theory parameters for the chemical sub-threshold and 1.5x threshold intensity stimuli were not evaluated, the statistical analyses were conducted independently for each intensity level between stimulus types. A paired sample *t*-test was conducted to compare the d_a_ between cold and mechanical stimuli of sub-threshold and 1.5x threshold intensity. The d_a_’s of both sub-threshold and 1.5x threshold intensity were not significantly different between the stimulus types (*p* > 0.05). On the other hand, a mixed-model analysis was conducted to compare the d_a_’s between the stimulus types of thresholds and 2× threshold intensity. The d_a_’s of the threshold intensity stimuli were not significantly different between the stimulus types [F (2, 70) = 2.988, *p* = 0.057], though the box plot showed a higher d_a_ for chemical stimuli in comparison to cold and mechanical stimuli (Figure 3). The d_a_’s of the 2× threshold intensity were not significantly different between stimulus types. A similar analysis for the A_z_ also showed similar comparisons as the d_a_.

**TABLE 3:** Average (±SE) d_a_ for all three stimulus types and stimulus intensities.

SDT Parameters	Stimulus intensity	Cold (non-noxious)	Mechanical (noxious)	Chemical (noxious)
Detectability (d_a_) (mean ± SE)	Sub-threshold	−0.15 ± 0.13	0.10 ± 0.14	NA
	Threshold	0.66 ± 0.12	0.68 ± 0.11	0.97 ± 0.12
	1.5× threshold	1.33 ± 0.17	1.57 ± 0.17	NA
	2× threshold	1.90 ± 0.17	2.08 ± 0.19	1.88 ± 0.16

### Within Stimulus Comparisons

#### Cold Stimulus

**FIGURE 4:**
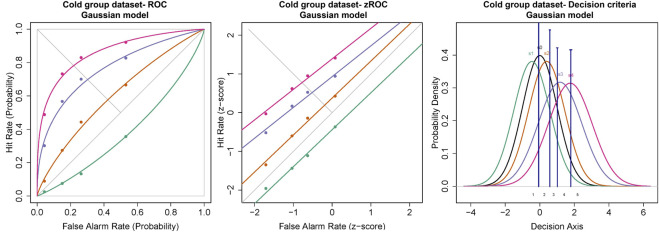
ROC and Gaussian distribution for the cold stimuli. The green line represents the sub-threshold stimuli followed by orange (threshold), purple (1.5× threshold), and pink (2× threshold). The black line (S_0_) in density functions represent the noise distribution.

**FIGURE 5:**
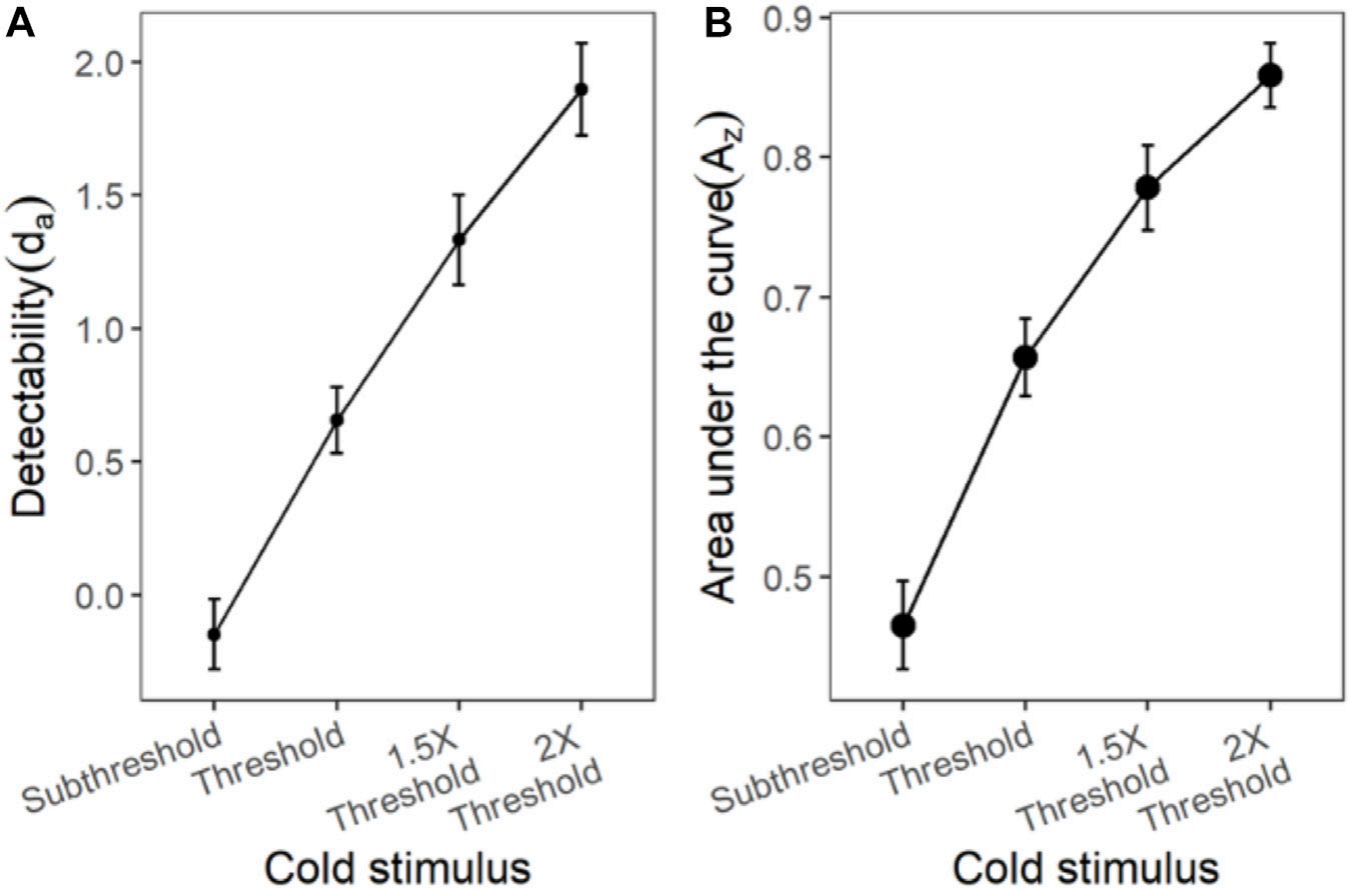
The d_a_ and A_z_ transducer functions for cold stimuli. Each horizontal axis is stimulus intensity and in the left-hand panel, the *y*-axis is detectability (d_a_) and in the right-hand panel, the *y*-axis is area under the curve (A_z_). The points are the means and error bars are the SE of the estimates.

The ROC curves plotted using the cumulated ratings showed a good separation in the d_a_ between the scaled stimulus intensities (Figure 4). The ROC curve of cold sub-threshold intensity stimuli was inverted, indicating a negative d_a_. The z-ROC curves for all stimuli were almost parallel to the chance line and only the z-ROC of the sub-threshold intensity stimuli was below the chance line similar to the ROC curve. The slopes of the supra-threshold z-ROC were less than 1, but the curves did not cross each other or other curves within the stimulus type. A mixed-model analysis was conducted to compare the d_a_ of the cold stimuli between the intensities. A significant main effect of stimulus intensity [F (3,130) = 29.91, *p* < 0.001] was observed for d_a_ between the cold stimulus intensities (Figure 5). The contrast analysis showed that the d_a_ of each intensity was significantly different from the other. Similarly, the analysis of the area under the curve was also found to be significantly different between the intensities [F (3, 94.96) = 129.91, *p* < 0.001].

#### Chemical Stimulus

**FIGURE 6:**
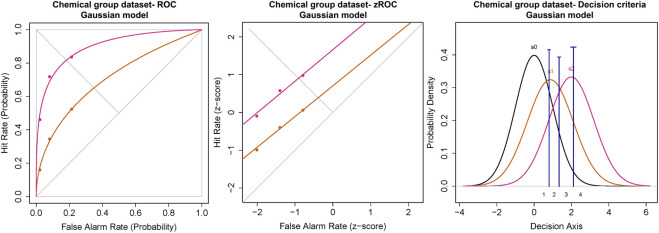
ROC and Gaussian distribution for the chemical stimuli. The orange line represents the threshold stimuli followed by pink (2x threshold). The density functions in black (s_0_) represent the noise distribution.

**FIGURE 7:**
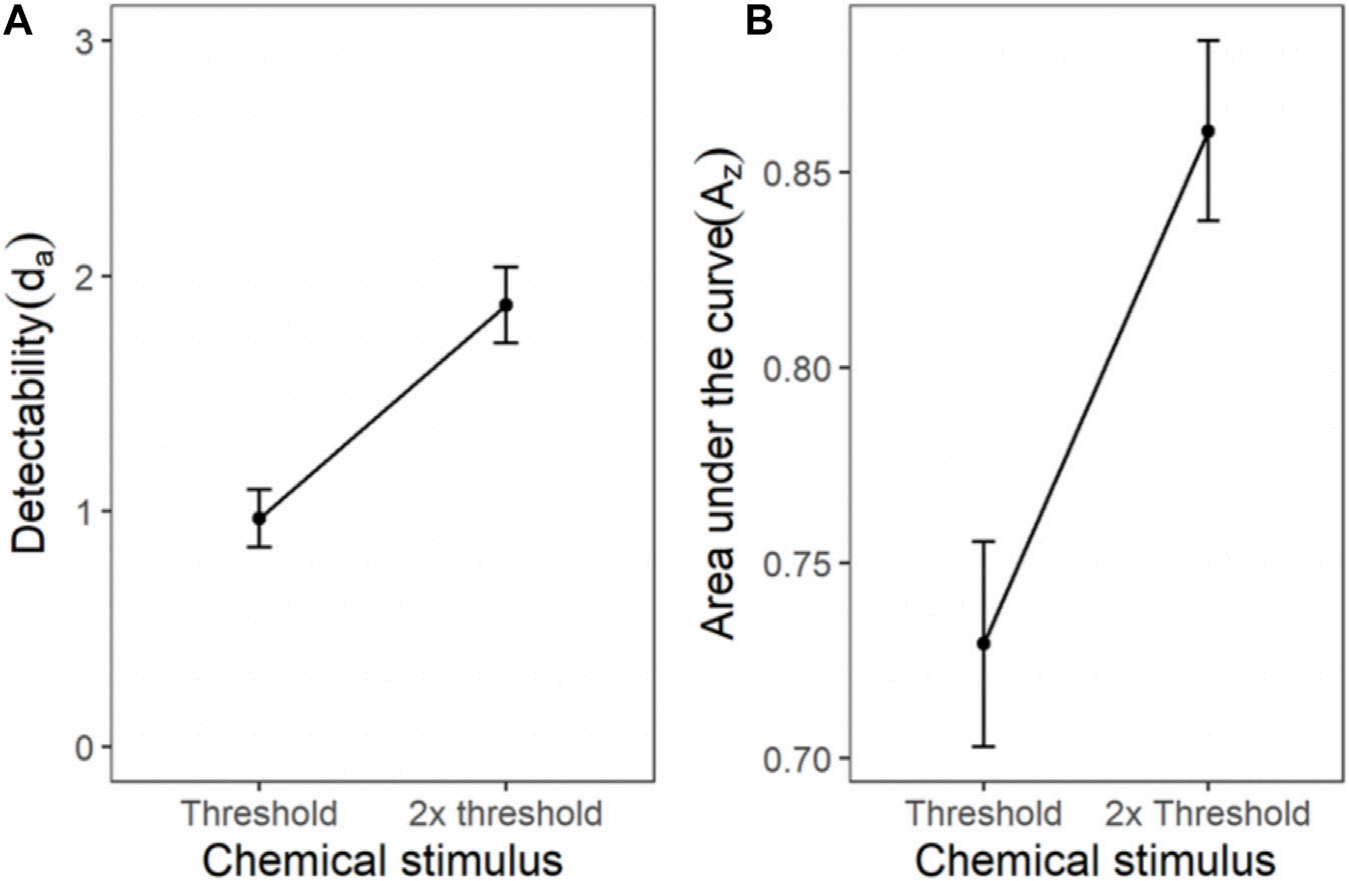
The transducer function for d_a_ and A_z_ of chemical threshold and 2x threshold stimuli. Each horizontal axis is stimulus intensity and in the left-hand panel, the *y*-axis is detectability (d_a_) and in the right-hand panel, the *y*-axis is area under the curve (A_z_). The points are the means and error bars are the SE of the estimates.

The ROC for the cumulated ratings of all participants showed good separation between the d_a_s of the threshold and 2x threshold intensity chemical stimuli (Figure 6). The slope of the z-ROC of the 2× threshold intensity stimuli was parallel to the chance line, whereas the slope was slightly less than 1 for threshold intensity stimuli. A paired sample *t*-test was conducted, and a significant difference was observed between the d_a_’s of the threshold (0.97 ± 0.12) and 2× threshold (1.88 ± 0.16) intensity stimuli; t (35) = −5.93, *p* < 0.001 (Figure 7). Similarly, the A_z_ was also significantly different between the two stimulus intensities [t (35) = −5.41, *p* < 0.001] (Figure 7).

#### Mechanical Stimulus

**FIGURE 8:**
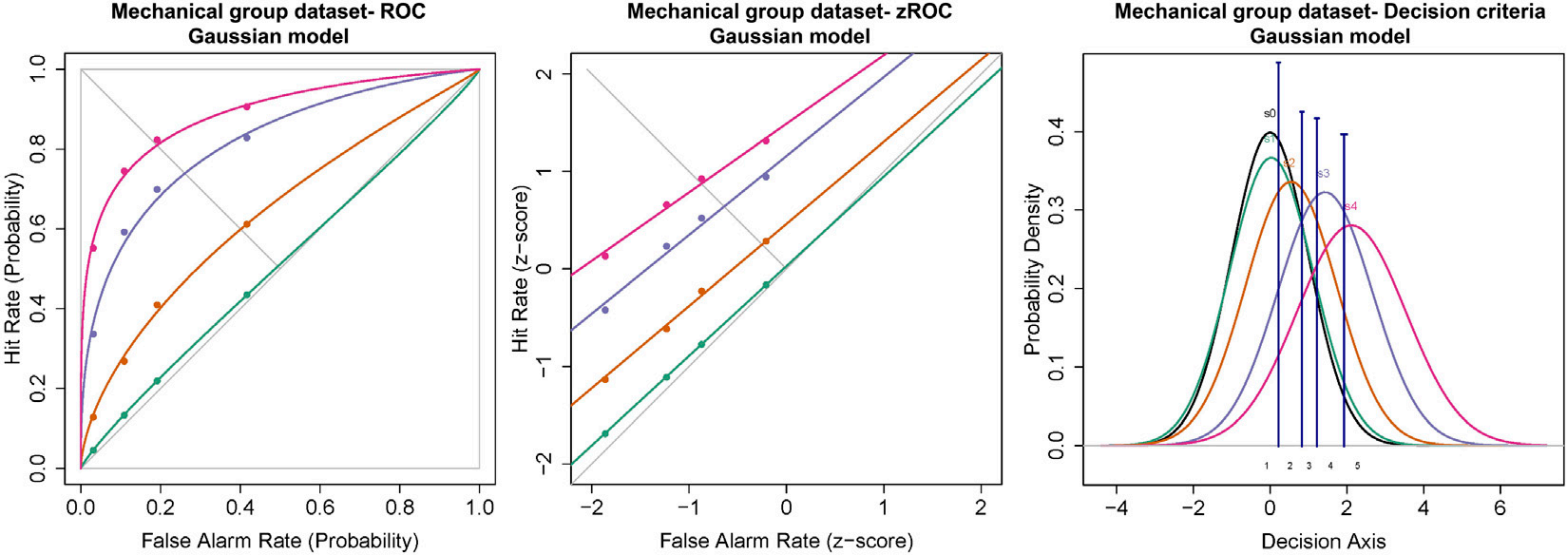
ROC, zROC, and Gaussian distribution for the mechanical stimuli. The green line represents the sub-threshold stimuli followed by orange (threshold), purple (1.5x threshold), and pink (2x threshold). The black line (S_0_) in density functions represent the noise distribution.

**FIGURE 9:**
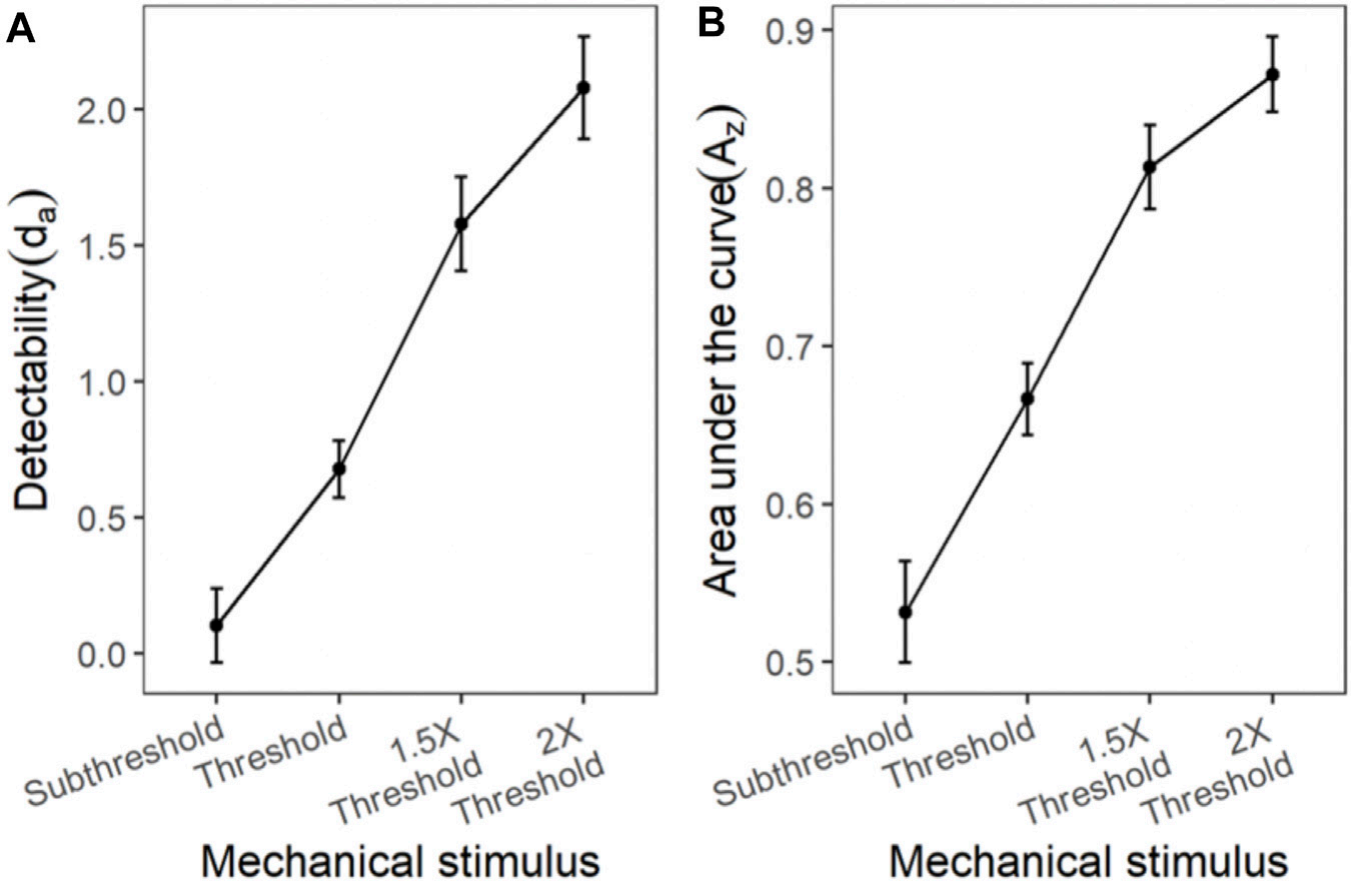
The transducer function for d_a_ and A_z_ of mechanical stimuli. Each horizontal axis is stimulus intensity and in the left-hand panel, the *y*-axis is detectability (d_a_) and in the right-hand panel, the *y*-axis is area under the curve (A_z_). The points are the means and error bars are the SE of the estimates.

Similar to the cold and chemical stimuli, there was good separation between the ROC curves of different stimulus intensities (Figures 8, 9). The slopes of z-ROC were less than one and the z-ROC of sub-threshold intensity crossed the chance line. The mixed-model analysis showed that the d_a_’s of the mechanical stimuli were significantly different between the intensities used in the experiment [F (3,100.92) = 66.46, *p* < 0.001] (Figure 9). A_z_ showed a similar significant main effect of the intensities [F (3,100.63) = 60.96, *p* < 0.001] (Figure 9).

#### Criterion

Both c and *lnβ* were analyzed in this experiment but only the results for *lnβ* are discussed due to the length of the manuscript.

#### Cold Stimulus Criterion lnβ

**FIGURE 10:**
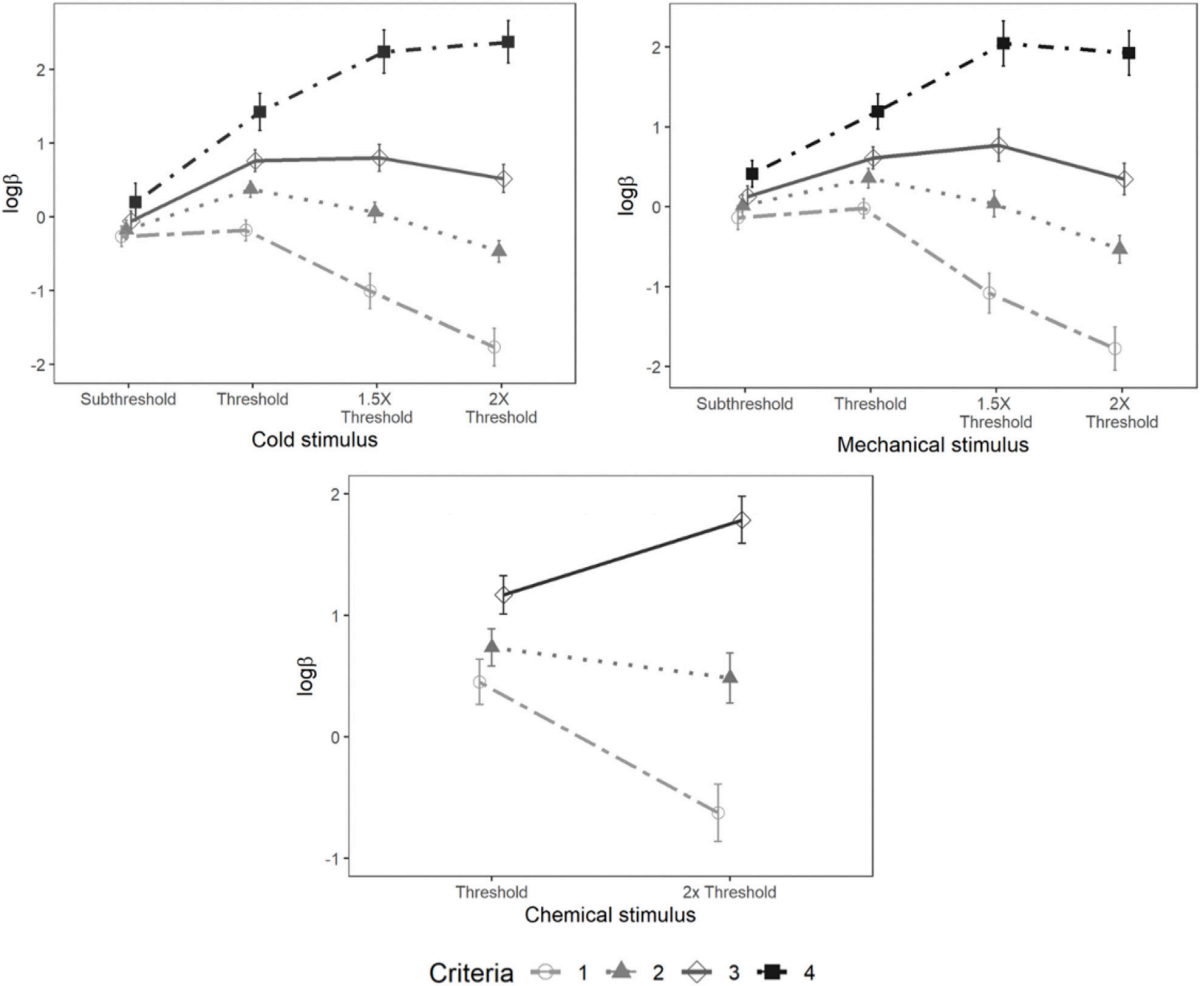
Loglikelihood ratio for cold, mechanical, and chemical stimulus. Each horizontal axis is stimulus intensity and vertical axis is *ln*β. The points are the means and error bars are the SE of the estimates. The lines indicate different criteria.

Mixed-model analysis of *lnβ* also showed a significant main effect of psychophysical criterion [F (3,95.94) = 15.34, *p* < 0.001] and stimulus intensity [F (3,104.83) = 32.50, *p* < 0.001]. A significant interaction was also observed between the psychophysical criterion and intensity [F (9,285.85) = 51.59, *p* < 0.001] (Figure 10).

#### Mechanical Stimulus Criterion lnβ

There were significant main effects of psychophysical criterion [F (3,105) = 49.44, *p* < 0.001] and stimulus intensity [F (3,101.64) = 7.56, *p* < 0.001] as well as a significant interaction between the stimulus intensity and psychophysical criterion [F (9,304.08) = 38.38, *p* < 0.001] (Figure 10).

#### Chemical Stimulus Criterion *lnβ*

A significant main effect of psychophysical criterion was observed [F (2,70) = 52.10, *p* < 0.001] along with a significant interaction between the stimulus intensities and psychophysical criterion [F (2,70) = 19.68, *p* < 0.001]. However, *lnβ* was not significantly different between stimulus intensities (Figure 10).

## Discussion

The primary purpose of this experiment was to determine the feasibility of conducting an MSDT experiment using painful and cooling pneumatic ocular surface stimuli. We have shown that SDT may be used in a yes/no experiment, but there were drawbacks, some of which might be overcome if multiple stimulus intensities and participants using multiple criteria were possible (Jayakumar and Simpson, 2020). We showed that this more complex experimental design was feasible: Participants were able to concentrate during the experiments and were very well-behaved sensory (A_z_ and d_a_) and criteria (here, *lnβ*) metrics were reliably derivable (Jayakumar and Simpson, 2020). Because of the results reported here, additional predictor variables related to patient anxiety and decision making could be studied and their effects on d’ and *lnβ* evaluated^1^.

There were several results indicating the internal validity of the data we found. Although this paper is primarily about the feasibility of signal detectability (and in signal detection theory, “thresholds” do not exist), the detection thresholds (used in deriving stimulus intensities for the MSDT experiments) obtained in this study were consistent with previous studies that measured corneal detection thresholds as a primary outcome measure (Feng and Simpson, 2003; Situ et al., 2008; BasuthkarSundarRao and Simpson, 2014; Jayakumar et al., 2015; Alabi and Simpson, 2020). In addition, the MSDT data for pneumatic stimuli used in this study followed the assumptions of SDT, which were evident in the ROC curves and Gaussian distributions reported in the results (Figures 4, 6, 8). The ROC curves obtained were well behaved (with low residuals for each ROC line) for all stimulus types and the curves (both in ROC and z-ROC space) for intensities within each stimulus type did not overlap, indicating independent detectabilities for the scaled intensities. The z-ROC curves were almost parallel to the chance (45°) line, indicating the adherence of the obtained data to 1) Gaussian assumptions and 2) approximately equal variance in basic signal detection theory. The d_a_s calculated were similar using both cumulated rating data method and the average of the individual detectabilities (Table 4). This similarity in the d_a_ between the two methods indicates that the group detectability can be computed either from individual d_a_’s or from group averaged d_a_’s for ocular surface stimuli scaled based on detection thresholds. These results collectively point to the feasibility and internal (and face) validity of using MSDT (with intensities scaled based on detection thresholds) in analyzing the OSSP of the pneumatic stimuli.

**TABLE 4:** Comparison of d_a_ obtained using cumulated and individual rating data.

Stimulus type	Stimulus intensity	d_a_ using cumulated rating data	Average d_a_ calculated from the d_a_ of each participant
Cold	Sub-threshold	−0.46	−0.15 ± 0.13
	Threshold	0.40	0.66 ± 0.12
	1.5× threshold	1.17	1.33 ± 0.17
	2× threshold	1.77	1.90 ± 0.17
Mechanical	Sub-threshold	0.03	0.10 ± 0.14
	Threshold	0.55	0.68 ± 0.11
	1.5× threshold	1.43	1.57 ± 0.17
	2× threshold	2.11	2.08 ± 0.19
Chemical	Threshold	0.87	0.97 ± 0.12
	2× threshold	1.99	1.88 ± 0.16

Another metric of experimental feasibility is the number of participants who could not complete the experimental protocol. It is not useful if a substantial proportion of participants cannot do the experiments, even if the data from (a smaller number of) participants are well behaved. Two participants could not complete all the experiments due to their high baseline detection thresholds, and three participants could not complete due to variable detection thresholds. Neither of these groups of participants could not be used because of the signal detection theory aspects of the experiments: They were excluded because of the preliminary results, so considering the complex and noisy nature of the OSSP system, the results were very promising and clearly indicate the feasibility of these study methods.

### Detection Theory Indices

The d_a_ (obtained from ROC using cumulated data) of all three stimulus types (the intensities of which were scaled based on their respective detection thresholds), showed a systematic increase with increase in the intensity of the stimuli. Such behavior of these transducer functions was, of course, expected: Similar increases in the average d_a_ have been observed in the transducer functions in other senses (e.g., vision) (Nachmias, 1972). In addition, in this experiment, although 2 of the 3 stimuli were nociceptive (mechanical and chemical), as is apparent in Figures 5, 7, 9, there were no differences between nociceptive and non-nociceptive transducer functions.

*lnβ* showed a relatively complex dependency on the psychophysical criterion and stimulus intensity, especially as participant criterion increased. This complexity is somewhat scientifically problematic, because, ideally, one might prefer bias metrics should be approximately independent of stimulus intensity and the interaction is a further complication. What this does, however, is highlight the problem with psychophysical methods that do not derive any criterion metric, such as traditional Fechnerian methods. Detection thresholds combine sensory and decision components, and they cannot be disambiguated. If, as we show in our experiment, there are complex relationships between intensity and criteria, then methods that cannot disentangle these 2 are more difficult to interpret.

### Detectability

Stimulus detectability may be derived in several ways: Using each hit rate (saying a stimulus was present when it was) and false alarm rate (saying a stimulus was present when it was not) the equations from individual/group data in the introduction may be used. Using collections of hit rate and false alarm rate for each criterion used, one might derive ROC curves from which detectability may also be derived. In this work there were consistent results that made it clear that it did not matter what approach was used. The transducer functions and the ROC curves all strongly pointed to the same conclusion that there was a clear separation of threshold scaled stimuli for both painful and cold corneal stimulation, again, pointing to the utility of a reliable SDT detection metrics being obtainable using the experimental design selected, as well as providing compelling evidence of the external validity of our data. The results are perfectly in line with several aspects of signal detection theory that predict how detectability scales with intensity and how criteria shift along ROC (iso-detection) curves.

We hypothesised that d_a_ derived using corneal pneumatic stimuli would be different between the intensities and also, on the basis of our earlier work (Tanelian and Beuerman, 1984), between the stimulus types (nocimetric and non-nocimetric). SDT proposes the sensory process as a continuous output, detectability, that is a function of the separation of a noise distribution and a signal-plus-noise distribution, unlike the threshold theory that defines the stimulus as always detectable once it crosses a threshold (and not detectable below threshold) (Wickens, 2010). The change in the detectability with stimulus intensity was evident in our experiment for each of the three types of stimuli, something reported previously in other senses, e.g., (Tanner and Swets, 1954; Stromeyer et al., 1982; Stromeyer et al., 1984). Since other ocular MSDT studies are not available for comparison within the ocular somatosensory system, the human response to similarly scaled stimulation might need to be examined indirectly. Alabi and Simpson (Alabi, 2018; Alabi and Simpson, 2019; Alabi and Simpson, 2020) observed a dose-effect increase in the autonomic responses such as redness, pupillary response, and accommodation for pneumatic stimuli. Situ et al. (Situ and Simpson, 2010) also reported an increase in the tearing response (using tear meniscus height measurement) and these taken together point to similar monotonic scaling of the human psychophysiological response to painful and cooling corneal stimulation. We and others have contributed reports of increases in ratings of attributes of ocular surface stimulation with increasing stimulus intensity in humans (Chen et al., 1995; Belmonte et al., 1999; Acosta et al., 2001; Feng and Simpson, 2004; Situ, 2010; Wu et al., 2015; Situ et al., 2019). Our detectability results, although with stimuli that are ‘circum-threshold’ or slightly suprathreshold are, therefore, in line with other work in humans that show physiological and perceptual responses to the stimuli we used without the complication of the effect of participants’ criteria.

Figure 2 shows that there is a systematic increase in d’ with increase in stimulus intensity. A cursory understanding might suggest that this is nothing more than suprathreshold scaling [say a manifestation of Stevens Power Law (Wickham and Henry, 2019)]. This is not that simplistic: In a suprathreshold scaling experiment, an observer reports some value (derived using magnitude estimation or another form of scaling) that matches the subjective (perceived) intensity of the stimulus. This has 2 components, an intensity component and a criterion component. SDT methods enable a separation of this scalar value into a vector with 2 pieces, the sensory and the decision component. Detectabilty is one of those components and is not “simply” related to a suprathreshold score, because it acknowledges (and is mathematically derived from) the experimental fact that it (d’) includes scores related to the ***absence*** as well as the ***presence*** of the stimulus. The interpretation that this then somehow is just the same as the suprathreshold scaling ignores another primary observation we made: The decision component ***also*** is a function of stimulus intensity (in a more complicated way as is shown by the interaction with stimulus intensity in Figure 9). Finally, we used a subthreshold stimulus for mechanical and cooling stimuli, that when using conventional suprathreshold methods could simply not be feasible since it would ***not*** be perceived by the observer for the majority of the stimulus presentations. Because of the multiple criterion method used, this extremely low stimulus intensity did not detract from what was feasible experimentally and so detectability and criteria metrics were derived as expected from SDT. Finally, it should be pointed out how badly behaved some suprathreshold scaling functions actually are, with many saturating and inverted perceived intensity vs. stimulus intensity functions, illustrating that suprathreshold scaling methods used do not always result in outcomes that are as might be predicted physiologically.

### Physiological Interpretations

These results were almost perfectly in accordance with signal detection theory. The basic physiological implications are therefore fairly direct. The distribution of firing frequency of quiescent sensory neurons is Gaussian and against that distribution, decisions about sensory stimulation are made–is the Gaussian distribution of firing frequency of the stimulated (ocular surface) neuron (or system of neurons) different from that when there is no noise. In the context of the effect of a drug on the eye that alters this process, there are a number of ways to affect the outcome. In the first place, the distribution of the noise could be altered, either by reducing spontaneous firing or changing (reducing) the variance of the noise distribution. In these instances, a criterion stimulus would be more detectible (something not necessarily desirable if the eye is already uncomfortable). If it were desirable to reduce the effect of a painful/unpleasant stimulus, the drug could affect the stimulated distribution by reducing the firing frequency or altering the firing frequency variability so that detectability was lowered. Another possibility is to alter the decision so that the detectability is unchanged, but the firing frequencies are interpreted in a more conservative way, say, so that the observer patient is either less likely to call a criterion stimulus a stimulus (i.e., report that it is absent) or be less certain about the presence of a painful/uncomfortable stimulus. This is not to say that different from the interpretation of work on placebos using signal detection theory (Clark, 1969; Rollman, 1977; Allan and Siegel, 2002). This, of course, would imply more central acting and not peripheral acting pharmacological activity. This dichotomy of action based on detection theory is in line with the model of pain processing being a combination of a sensory/intensity dimension with an affective motivational modulation aspect (Williams and Craig, 2016). The simple clinical relevance of both the sensory and criteria metrics is however yet to be explored, as this work was a test of basic detection theory concepts. In particular, the measurement of bias is important, but more testing is also needed to evaluate ways to control/manipulate bias before it can be routinely applied in clinical measurements.

### Limitations

There were a few instruments and psychophysical method related limitations in this experiment. The instrument related limitations were the Belmonte esthesiometer’s stimulus range and the time taken to prepare the chemical stimuli. The Waterloo Belmonte esthesiometer has a reliable stimulus flow rate range of 10–200 ml/min. In addition, the maximum concentration of added CO_2_ in chemical stimuli can be only 100%. Since the MSDT experiment has stimuli of intensities at the detection threshold, as well as sub-threshold (0.5x detection threshold) and supra-threshold (1.5x and 2x detection threshold) levels, limitations arose when the scaled intensities fell outside the stimulus range available. For example, if the participant had a high chemical detection threshold of 70%, both supra-threshold intensities (105 and 140%) are outside the physical range of concentrations possible. Similarly, if the participant had a high mechanical detection threshold of 115 ml/min, the 2× supra-threshold (230 ml/min) stimuli would be outside the stimulus range available from the Waterloo Belmonte instrument. The 2 of 41 participants with these high detection thresholds were excluded from the experiment.

Another limitation of our esthesiometer was the time taken between chemical stimuli to purge the esthesiometer delivery tubes for each subsequent stimulus. To keep each stimulus-type experiment approximately the same duration, we used fewer chemical intensities and fewer chemical trials and were then able to keep an approximately constant stimulus probability across nocimetric and non-nocimetric stimulus types. The number of ratings were also reduced to minimize rating categories with no responses. A therefore unavoidable (obvious) consequence of these changes was observed in the analysis when detection indices were compared between stimulus types due to the difference in the number of ratings and number of intensities between stimulus types. Although complicating the inferences that could be made because of the unbalanced design, this did not influence our ability to compare stimulus types, however.

The training was provided to participants to familiarize them with the experimental set-up, the audio prompts during the experiment, and how to use the response button box. Participants were also instructed about different intensities before the MSDT trials. Because of the inclusion of (separate) anxiety measurement (Wickham, 2016) between experiments, feedback was not provided after each response; part of the experiment was to monitor anxiety change during the experiment. Future work may be needed to evaluate the exact effect of more extensive training and the effects of perceptual learning on the sensory and decision metrics used in this experiment as well as whether feedback would affect the results reported here.

In conclusion, we showed 1) MSDT is feasible for analyzing ocular surface sensory processing and 2) detectability and bias may be reliably extracted when using pneumatic stimuli. Specifically, detectability (d_a_) of scaled threshold intensities systematically increases and the bias psychophysical criterion (*ln*β) systematically varies with stimulus intensity. In humans, during ocular surface processing of noxious and non-noxious pneumatic stimulation, detectability and criteria vary systematically with stimulus intensity, a result that cannot be derived using classical psychophysics.

## Data Availability

The raw data supporting the conclusions of this article will be made available by the authors, without undue reservation.

## Footnotes

1 Ocular Surface Sensory Processing and Signal Detection Theory, September 2021, A PhD thesis presented by Varadharajan Jayakumar to the University of Waterloo.
